# Emergence of two prion subtypes in ovine PrP transgenic mice infected with human MM2-cortical Creutzfeldt-Jakob disease prions

**DOI:** 10.1186/s40478-016-0284-9

**Published:** 2016-02-05

**Authors:** Jérôme Chapuis, Mohammed Moudjou, Fabienne Reine, Laetitia Herzog, Emilie Jaumain, Céline Chapuis, Isabelle Quadrio, Jacques Boulliat, Armand Perret-Liaudet, Michel Dron, Hubert Laude, Human Rezaei, Vincent Béringue

**Affiliations:** INRA (Institut National de la Recherche Agronomique), UR892, Virologie Immunologie Moléculaires, F-78350 Jouy-en-Josas, France; Neurobiology Laboratory, Biochemistry and Molecular Biology Department, Hôpitaux de Lyon, Lyon, France; University of Lyon 1, CNRS UMR5292, INSERM U1028, BioRan, Lyon, France; Neurology Department, Centre Hospitalo-Universitaire, Bourg en Bresse, France

**Keywords:** Prion, CJD, Mutation, Sporadic, Transgenic mice

## Abstract

**Introduction:**

Mammalian prions are proteinaceous pathogens responsible for a broad range of fatal neurodegenerative diseases in humans and animals. These diseases can occur spontaneously, such as Creutzfeldt-Jakob disease (CJD) in humans, or be acquired or inherited. Prions are primarily formed of macromolecular assemblies of the disease-associated prion protein PrP^Sc^, a misfolded isoform of the host-encoded prion protein PrP^C^. Within defined host-species, prions can exist as conformational variants or strains. Based on both the M/V polymorphism at codon 129 of PrP and the electrophoretic signature of PrP^Sc^ in the brain, sporadic CJD is classified in different subtypes, which may encode different strains. A transmission barrier, the mechanism of which remains unknown, limits prion cross-species propagation. To adapt to the new host, prions have the capacity to ‘mutate’ conformationally, leading to the emergence of a variant with new biological properties. Here, we transmitted experimentally one rare subtype of human CJD, designated cortical MM2 (129 MM with type 2 PrP^Sc^), to transgenic mice overexpressing either human or the VRQ allele of ovine PrP^C^.

**Results:**

In marked contrast with the reported absence of transmission to knock-in mice expressing physiological levels of human PrP, this subtype transmitted faithfully to mice overexpressing human PrP, and exhibited unique strain features. Onto the ovine PrP sequence, the cortical MM2 subtype abruptly evolved on second passage, thereby allowing emergence of a pair of strain variants with distinct PrP^Sc^ biochemical characteristics and differing tropism for the central and lymphoid tissues. These two strain components exhibited remarkably distinct replicative properties in cell-free amplification assay, allowing the ‘physical’ cloning of the minor, lymphotropic component, and subsequent isolation in ovine PrP mice and RK13 cells.

**Conclusions:**

Here, we provide in-depth assessment of the transmissibility and evolution of one rare subtype of sporadic CJD upon homologous and heterologous transmission. The notion that the environment or matrix where replication is occurring is key to the selection and preferential amplification of prion substrain components raises new questions on the determinants of prion replication within and between species. These data also further interrogate on the interplay between animal and human prions.

**Electronic supplementary material:**

The online version of this article (doi:10.1186/s40478-016-0284-9) contains supplementary material, which is available to authorized users.

## Introduction

Mammalian prions are proteinaceous pathogens responsible for a broad range of fatal neurodegenerative diseases in humans and animals [20]. Prions are primarily formed of macromolecular assemblies of PrP^Sc^, a misfolded, ß-sheet enriched form of the ubiquitously expressed, plasma membrane-anchored, variably N-glycosylated and α-helix rich, host-encoded prion protein PrP^C^ [49]. This change is based on the self-sustained transfer of a structural information from the PrP^Sc^ conformer in the prion state to PrP^C^, presumably through a seeding-polymerization process [19]. Within defined host species, PrP^C^ can transconform in multiple prion variants or strains, differing in their PrP^Sc^ conformations at the level of the tertiary and/or quaternary structure, in their biological properties and in their relative capacity to replicate in cell lines or tissues [5, 8, 14, 21, 37]. Prions can propagate within and between species, as exemplified by the emergence of human variant Creutzfeldt-Jakob disease (vCJD) through dietary exposure to prions responsible for the bovine spongiform encephalopathy (BSE) epidemics in cattle [62]. Within defined species, prions can also form sporadically. In humans, the incidence of sporadic Creutzfeldt-Jakob disease (sCJD) ranges between 1 and 2 cases per million and per year, and affects mainly elderly people [62]. Atypical BSEs (L-type and H-type) and atypical scrapie Nor98 are thought to develop spontaneously in aged ruminants [8].

Characterizing PrP^Sc^ electrophoretic pattern following limited digestion with proteinase K (PK) can allow differentiation of prion strains. For sCJD, methionine/valine (M/V) polymorphism at codon 129 of the gene encoding PrP and the migration pattern and relative glycoform abundance of PK-resistant PrP^Sc^ (PrP^res^) allow the definition of different molecular subtypes [28, 45, 57]. These subtypes exhibit specific clinical and neuropathological features [28, 45]. The most common form of sCJD is associated with the presence of type 1 (T1) PrP^res^ and homozygosity for methionine at codon 129. The unglycosylated band of T1 PrP^res^ migrates at 21 kDa in SDS-PAGE gels, and monoglycosylated forms predominate over diglycosylated ones. Rare forms of MM sporadic CJD with a Type 2 (T2) PrP^res^ type, - the unglycosylated form of which migrating at 19 kDa -, have been diagnosed. These forms are further subclassified as cortical and thalamic variants [40, 45, 51]. The cortical variant is distinguished from all other sporadic forms by the absence of experimental transmission to knock-in mouse models expressing human PrP at physiological levels [10, 30, 40].

Mice transgenic for PrP have been instrumental in deciphering prion strain diversity and in modeling experimentally the so-called species or transmission barrier that limits prions interspecies transmission (for review [8]). In essence, such mice are generated to express specific sequences from mammalian PrP^C^ on a mouse PrP-ablated background, and are inoculated with prions. Analyzing the clinical outcome, attack rate and the presence of PrP^Sc^ in brain and peripheral tissues where prion replication can occur [5, 27] allows the establishment of whether cross-interactions between host PrP^C^ and invading PrP^Sc^ structural landscapes are possible with regard to prion conversion. The conformational hypothesis [21, 60] posits that PrP^C^ can adopt a limited portfolio of conformations in the PrP^Sc^ state, due to structural constraints in its amino acid backbone. If the infecting PrP^Sc^ conformation(s) is within the portfolio of possible conformations, cross-species transmission will occur. If not, the transmission barrier will be high, and can lead to an abrupt change in prion strain biological properties [2, 4, 5, 9, 13, 29, 38, 46, 47, 54], a phenomenon referred to as a ‘mutation’. Whether the newly emerging strain is selected from an ensemble of pre-existing PrP^Sc^ conformations in the original inoculum (‘quasi-species’) or is generated *ex abrupto* remains difficult to determine. A high transmission barrier does not lead systematically to prion strain ‘mutation’, as highlighted by the remarkable ability of classical BSE prions to retain their biological properties, despite intermediate passage to a range of different hosts [12, 15, 35].

Here, we studied the strain biological properties of one rare subtype of sporadic CJD prions, the cortical MM2-form, upon transmission to either human or ovine PrP transgenic mice.

## Materials and methods

### Ethics statement

All animal experiments were approved by the Local Ethics Committee of the authors’ institution (Comethea; permit number 12/034). Human tissues samples were selected from the tissue bank of the French National Neuropathology Network for CJD on the basis of the availability of autopsy-retained frozen brain material and informed consent from patient’s relatives for autopsy and research use, according to the French regulation (L.1232-1 to L.1232-3, Code de la Santé Publique).

### Transgenic mouse lines

The human PrP tg650 and ovine PrP tg338 lines have been described previously [5, 7, 34]. These lines are homozygous with approximately 6-fold and 8-fold over-expression of human PrP^C^ (M_129_ allele) and ovine PrP^C^ (V_136_R_154_Q_171_ allele) in brain, respectively.

### MM2-sCJD transmission and titration

To avoid any cross-contamination, a strict protocol based on the use of disposable equipment and preparation of all inocula in a class II microbiological cabinet was followed. A cerebellum extract was used as source of sporadic CJD, cortical MM2 subtype. This sample has a T2 specific PrP^res^ molecular profile. The tissue extract was prepared as 10 % w/v homogenate in 5 % w/v glucose with a Precellys (Ozyme, Montigny-le-Bretonneux, France) for inoculation into tg650 and tg338 mice. Twenty microliters were inoculated intracerebrally in the right hemisphere to groups of individually identified 6-8 week-old tg650 or tg338 mice, at the level of the parietal cortex. The first mouse succumbing with disease was used for subpassaging. The brain and spleen (when specified) of this mouse were collected with distinct, disposable tools, homogenized at 20 % w/v in 5 % glucose and reinoculated intracerebrally at 10 % w/v.

For endpoint titration, starting from 10 % w/v brain homogenate (‘undiluted’ material), serial 10-fold dilutions of brain homogenates were prepared in 5 % w/v glucose containing 5 % w/v bovine serum albumin. Twenty microliters of each dilution were immediately inoculated into individually identified 6-8-week-old tg650 or tg338 recipient mice by intracerebral route. Animals were supervised daily for the appearance of neurological signs associated with the development of a prion disease. Animals at terminal stage of disease or at end of life were euthanized. The brains and spleens of all animals were analyzed for PrP^res^ content. The number of prion-positive mice was used to establish, by the Spearman-Kärber method, the number of prion infectious units per gram of tissue leading to median mouse infection (ID_50_ per gram).

### Protein misfolding cyclic amplification (PMCA)

Mouse brain lysates from healthy tg338 mice were used as the substrate for tg650-passaged MM2-sCJD and tg338-adapted MM2-sCJD prion seeds. One round of PMCA, except indicated otherwise, was performed as previously described [41], in a microplate format, on a Q700 sonicator (Delta Labo, Colombelles, France), consisting in 96 cycles of 30 s of sonication at 200- to 220-W power followed by 29 min 30 s of incubation at 37 °C.

### Cell culture

RK13 cells and the Rov P2FJ6 clone expressing constitutively the VRQ allele of ovine PrP (tg2 construct; [59]) were routinely cultured in Opti-MEM, derived of the Eagle's minimal essential medium (opti-MEM; Gibco), supplemented with 10% fetal calf serum and antibiotics (penicillin, streptomycin), as previously described [23]. Cells were cultured at 37°C in a humidified 5 % CO_2_ atmosphere in a cell culture incubator. Cell monolayers in 12-well plate were exposed to brain or spleen homogenates from tg338 mice infected with ovine MM2-sCJD prions diluted in opti-MEM. Cells were exposed to 20 μl of 20 % brain homogenate or 40 μl of 10 % spleen homogenate diluted in 1 ml of fresh medium. One week later, cells were transferred to one 25-cm2 flask for growing. Each week (i.e. one passage), the cells were then split at the ¼ dilution in two flasks, one for further cultivation, one for PrP^res^ content analysis, as previously described [58]. Dissociation of the cells was made with a cell-dissociating buffer (Sigma).

### Immunoblot analyses

PrP^res^ was extracted from 20 % (wt/vol.) tissue homogenates with the Bio-Rad TeSeE detection kit, as previously described [4]. Briefly, aliquots were digested with proteinase K (200 μg/ml final concentration) for 10 min at 37 °C before B buffer precipitation and centrifugation at 28,000 × g for 15 min. Pellets were resuspended in sample buffer, denatured, run on 12 % Bis/Tris gels (Bio-Rad), electrotransferred onto nitrocellulose membranes, and probed with 0.1 μg/ml biotinylated anti-PrP monoclonal antibody Sha31 antibody (human PrP epitope 145-152, [24]) or with 0.1 μg/ml anti-PrP 12B2 antibody (human PrP epitope 89-93, epitope, [33]) and followed by streptavidin conjugated to horseradish peroxidase (HRP) or by HRP conjugated to goat anti-mouse IgG1 antibody (1/20 000 final dilution), respectively . Immunoreactivity was visualized by chemiluminescence (GE Healthcare). The size and relative amounts of PrP^res^ glycoforms were determined by the use of GeneTools software after acquisition of chemiluminescent signals with a GeneGnome digital imager (Syngene, Frederick, MD).

Enzymatic deglycosylation was performed on denatured PrP^res^ with 1,000 U of recombinant PNGase (New England BioLabs, Evry, France) for 2h at 37 °C in 1 % Nonidet P40 and the proprietary buffer. The deglycosylated proteins were then precipitated with three volumes of cold acetone and resuspended in Laemmli sample buffer for western blot analysis as described above.

### Histoblot analyses

Brains were rapidly removed from euthanized mice and frozen on dry ice. Cryosections were cut at 8-10 μm, transferred onto Superfrost slides and kept at -20 °C until use. Histoblot analyses were performed as described [36], using the 12F10 anti-PrP antibody (human PrP epitope 142-160, [31]). Analysis was performed with a digital camera (Coolsnap, Photometrics) mounted on a binocular glass (SZX12, Olympus). The sections presented are representative of the analysis of three brains samples.

## Results

### Description of the MM2-sCJD case

**Fig. 1:**
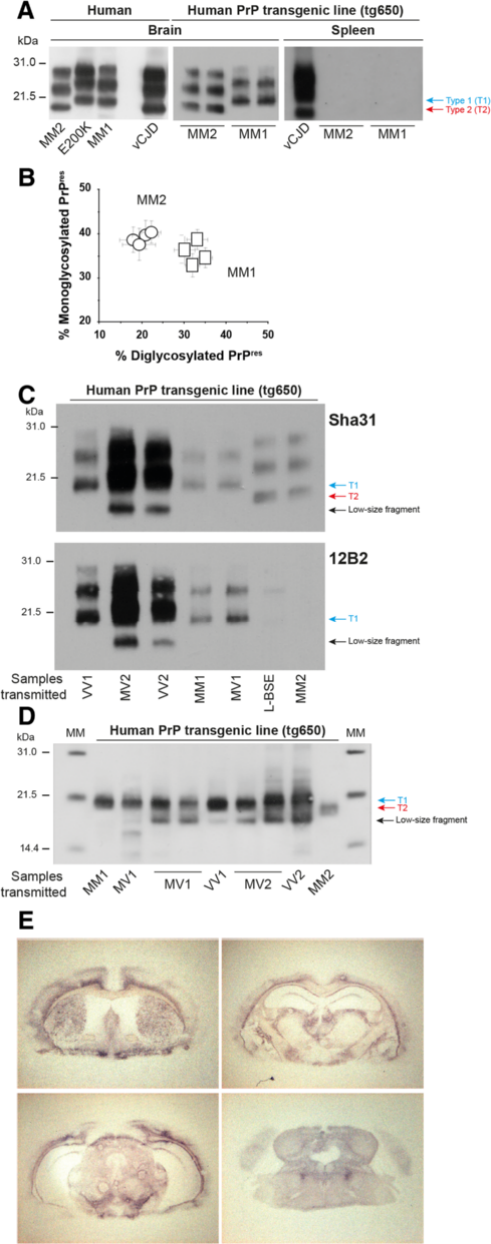
Biochemical and histopathological strain phenotype of MM2-sCJD prions in human PrP mice. (**a**) Electrophoretic pattern of cortical MM2-sCJD prions in human brain and in human PrP mouse (tg650) brains and spleens. Tissue homogenates were subjected to western blot analyses after limited proteinase K digestion. Blots were probed with Sha31 antibody. Other human prion sources (MM1-sCJD, E200K familial CJD and variant CJD) are shown as controls. The equivalent of 0.5 and 2 mg of brain and spleen tissue was loaded on the SDS-PAGE gel. Red and blue arrows denote the unglysosylated bands of PrP^res^ migrating at 21 kDa (T1) and 19 kDa (T2), respectively. Molecular masses (MM) of protein standards are indicated in kilodaltons. **(b)** Ratio of diglycosylated and monoglycosylated PrP^res^ species in the brains of tg650 mice following serial transmission (4 passages) of MM2-sCJD (circles) and MM1-sCJD (squares) prions (data plotted as means ± SEM, n = 6 mice analyzed at each passage). **(c)** Western blot analysis of PrP^res^ in the brain of tg650 mice infected with MM2-sCJD prions, after blotting with Sha31 antibody (top) or 12B2 antibody specific for Type 1 PrP^res^ (bottom). The banding patterns observed on transmission of other CJD subtypes and atypical L-BSE (which exhibits also a T2 signature, [4]) are shown for comparison. The equivalent of 1 mg (Sha31) and 7 mg (12B12) tissue were run on the SDS-PAGE gels for MM2-sCJD and L-BSE infected brains. The equivalent of 1 mg (Sha31, VV1, MM1, MV1; 12B2, MV2, VV2), 0.5 mg (Sha31 MV2, VV2), 2 mg (12B2, VV1, MM1, MV1) were loaded for the other samples. Note the presence of low-size PrP^res^ fragments in the brain of tg650 mice infected with MV2 and VV2 sCJD sources (black arrow). (**d**) Western blot analysis of PrP^res^ in the brain of tg650 mice infected with MM2-sCJD prions, after deglycosylation by PNGase F. Blots were probed with Sha31. Other CJD subtypes are shown for comparison. The equivalent of 0.2 mg of brain tissue (MM1, MM2), 0.1 mg (MV1, VV1) and 0.05 mg (MV2, VV2) were run on the SDS-PAGE gels. MM: molecular mass standards. (**e**) Regional distribution of PrP^res^ in the brain of tg650 mice infected with MM2-sCJD prions, by representative histoblots in 4 different antero-posterior sections. See [7] for comparison with transmission of MM1-sCJD prions. Histoblots were probed with 12F10 anti-PrP antibody

The MM2-sCJD brain source used in the study is a cortical variant, with a rapid clinical evolution. At the age of 62, the patient, who had comorbidities of diabetes, arterial hypertension and coronopathy, started to complain of memory loss and gait instability. He was admitted at the hospital, and neurological examination demonstrated dysphonia, cerebellar ataxia, confusion and cognitive impairment. The clinical picture rapidly worsened; 4 months later, he had dramatic cognitive impairment indicative of dementia and many falls backwards. MRI revealed unspecific leucopathy and one hypersignal in the right sylvian area. One month later, the patient was addressed to the emergency Unit for left hemiparesis, mutism and oppositional hypertonia. CSF analysis was positive for 14.3.3 protein. The EEG was negative. There was no pyramidal syndrome. There were myoclonia of the face. The situation evolved to absolute mutism and clonia of the face and of the left superior member. The EGG became periodic. The patient died 6 months after the onset of symptoms. There was no mutation in the *PRNP* gene. The patient was homozygous for methionine at PrP codon 129. At the autopsy, using the 3F4 anti prion antibody, PrP^Sc^ was detected in the cerebellum, in the striatum and in the frontal cortex by western-blot and immunohistochemistry. PrP^res^ exhibited a T2 electrophoretic pattern in each of these 3 cerebral areas, with predominance of monoglycosylated species and migration of the unglysosylated form at 19 kDa. This pattern was distinct from that characteristic of MM1-sCJD or vCJD (Fig. 1a).

### Faithful propagation and unique strain properties of MM2-cortical sCJD prions in human PrP mice

MM2-sCJD prions were inoculated intracerebrally to transgenic mice expressing the Met_129_ allele of human PrP (tg650 line [7]). All the inoculated mice succumbed with typical clinical signs of prion disease, with a mean incubation time of approximately 280 days. Four serial passaging, - including with cloned material -, produced no major change of the incubation time (Table 1).

**Table 1:** Serial transmission of MM2-cortical sCJD to mice expressing human or ovine PrP

Inoculum	Passage	Mean incubation time^b^ (n/n_0_)	
		tg650 (human PrP)	tg338 (ovine PrP)
MM2-sCJD	1	278 ± 6 (6/6)	557 ± 11 (6/7)
	2	274 ± 4 (8/8)	84 ± 1 (6/6)
	3	280 ± 4 (12/12)	81 ± 2 (6/6)
	4	268 ± 4 (8/8)^a^	78 ± 2 (7/7)
	5	nd	80 ± 1 (6/6)

n/n_0_: number of mice with neurological disease and positive for PrP^res^ in the brain by immunoblotting/number of inoculated mice

^a^Subpassage after cloning by end-point titration

^b^Days ± SE of the mean.

nd: not done

After each passage, the nervous and lymphoid tissues of the diseased mice were examined for the presence of PrP^res^, by immunoblotting. PrP^res^ was readily detected in the brains of all the mice analyzed. A typical T2 banding pattern, characterized by aglycosyl PrP^res^ migrating at 19 kDa and prominent monoglycoform species was observed (Fig. 1a-b), as in the human MM2-cortical case. This signature was conserved on subsequent passage and differed from that observed after transmission of the other sCJD subtypes to tg650 mice, as shown for comparison. Those elicited accumulation of PrP^res^ with a T1 signature alone (MM1) or concomitant with the presence of low-size fragments in variable amounts (MV1, VV1, VV2, MV2, Fig. 1a-d; Additional file 1: Figure S1). Only serial transmission of L-type BSE prions to tg650 mice produced a T2 signature in the brain (shown as T2 control in Fig. 1c, [4, 6]).

A number of PrP^res^ typing studies revealed the co-existence of T1 and T2 PrP^res^ in the brain of a variable percentage of sCJD cases [16, 48, 53, 57]. Analysis of PrP^res^ pattern in the brain of MM2-sCJD inoculated tg650 mice, either by using the 12B2 anti-PrP antibody that specifically recognizes T1 PrP^res^ or after deglycosylation failed to detect the T1 isoform (Fig. 1c-d).

PrP^res^ was not detectable in the spleens of tg650 mice inoculated with MM2-sCJD prions (Fig. 1a, n = 15 analyzed), at variance with vCJD (Fig. 1a and [7]).

Assessment of the neuroanatomical distribution of PrP^res^ by histoblotting revealed that MM2-sCJD prions deposited in the dorsal and habenular thalamic nuclei, in the optic tract, in the cingulum, in the external capsule, in the lateral hypothalamic area and in the trigeminal nuclei of tg650 brain (Fig. 1e).

After three passages in tg650 mice, MM2-sCJD prions were cloned by end-point dilution, as classically done [50], by inoculating serial 10-fold dilutions of infected brain material at terminal stage of disease to a cohort of tg650 mice. Based on clinical signs and presence of PrP^res^ in the brain, a 100% attack rate was observed until the 10^−4^ dilution. The limiting dilution established at 10^−6^ (Table 2). Applying the Spearman-Kärber method to the number of animals positive at each dilution provided a provisional infectious titer of 10^8.1^ intracerebral tg650 mouse ID_50_ U/g brain (ID_50_ IC in tg650/g), a value 50 to 100-fold lower than that calculated for MM1-sCJD and vCJD, respectively [7, 26].

**Table 2:** Endpoint titration of serially passaged MM2-sCJD prions in human or ovine PrP mice

Inoculum	Dilution	Mean incubation time^a^ (n/n_0_)	
		tg650 (human PrP)	tg338 (ovine PrP)
MM2-sCJD^b^	10^−4^	351 ± 6 (5/5)	nd
	10^−5^	441 ± 51 (3/5)	163 ± 10 (6/6)
	10^−6^	424; 501 (2/6)	227 ± 22 (4/6)
	10^−7^	>600 (0/6)	> 500 (0/6)
	10^−8^	>600 (0/6)	>500 (0/6)

n/n_0_: number of mice with neurological disease and positive for PrP^res^ in the brain by immunoblotting/number of inoculated mice

^a^Days ± SE of the mean.

^b^MM2-sCJD iteratively passaged in either tg650 or tg338 mice.

nd: not done

Collectively, these results indicate that cortical MM2-sCJD prions propagated with no transmission barrier in human PrP tg650 mice. The biological strain phenotype observed in these mice was unique amongst the other sCJD or vCJD sources transmitted so far.

### Isolation of 2 prion strain types with preferential tissue tropism on serial transmission of MM2-sCJD to ovine PrP mice

The same human source of MM2-sCJD was serially transmitted by intracerebral route to mice expressing the VRQ allele of ovine PrP (tg338 line; [34]), so as to assess both the cross-species transmission capacity of MM2-sCJD prions and the convertibility of ovine PrP^VRQ^ by foreign prions. On primary passage, six out of the seven inoculated mice developed a neurologic disease, with a mean incubation time of 557 ± 11 days, suggesting that propagation onto the ovine PrP sequence has occurred readily. On second passage, the mean incubation was dramatically shortened to approximately 80 days, a value that remained stable over 4 subpassages (Table 1). This abrupt change in the incubation time between the 1^st^ and the 2^nd^ passage is consistent with the isolation of a shorter incubation period variant [2, 4, 5, 8, 9, 13, 29, 46, 47, 54].

**Fig. 2:**
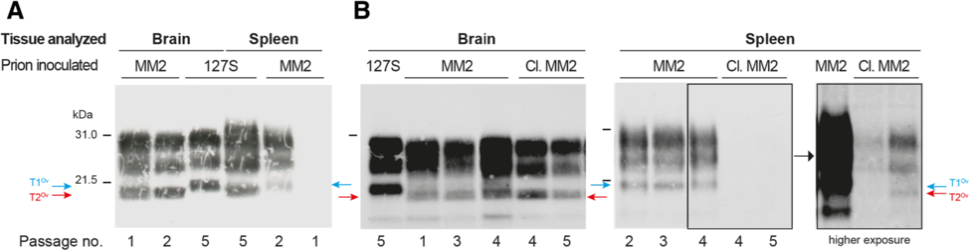
Banding patterns of PrP^res^ in the brain and spleen tissue of ovine PrP mice infected with MM2-sCJD prions. **(a)** PrP^res^ banding patterns in the brain and spleen tissue of ovine PrP mice (tg338 line) over two serial passages of MM2-sCJD (MM2). Note the different patterns in brain and spleen, which were designated T2^Ov^ (red arrow) and T1^Ov^ (blue arrow), respectively. These patterns can be compared with those observed after inoculation of 127S scrapie prions, a strain with a 21 kDa signature in brain and spleen [34]. (**b**) Immunoblots showing PrP^res^ pattern in the brain and spleen of tg338 mice over serial passage of uncloned MM2-sCJD (MM2) and cloned (Cl.) MM2-sCJD prions. Note the low levels of T2^Ov^ PrP^res^ in the spleens of tg338 mice inoculated with cloned MM2-sCJD prions, which are only visible on overexposed gels. The equivalent of 1 and 2 mg of tg338 brain and spleen tissue was loaded on the SDS-PAGE gels. The blots were probed with Sha31 antibody

PrP^res^ was detected by western blot in the brains of all diseased mice. A single banding pattern was observed on primary and subsequent passage. This pattern was designated T2^Ov^, as it resembled that of MM2-sCJD before and following passage in tg650 mice with regard to aglycosyl PrP^res^ migrating at ~19 kDa (Fig. 2a-b).

**Fig. 3:**
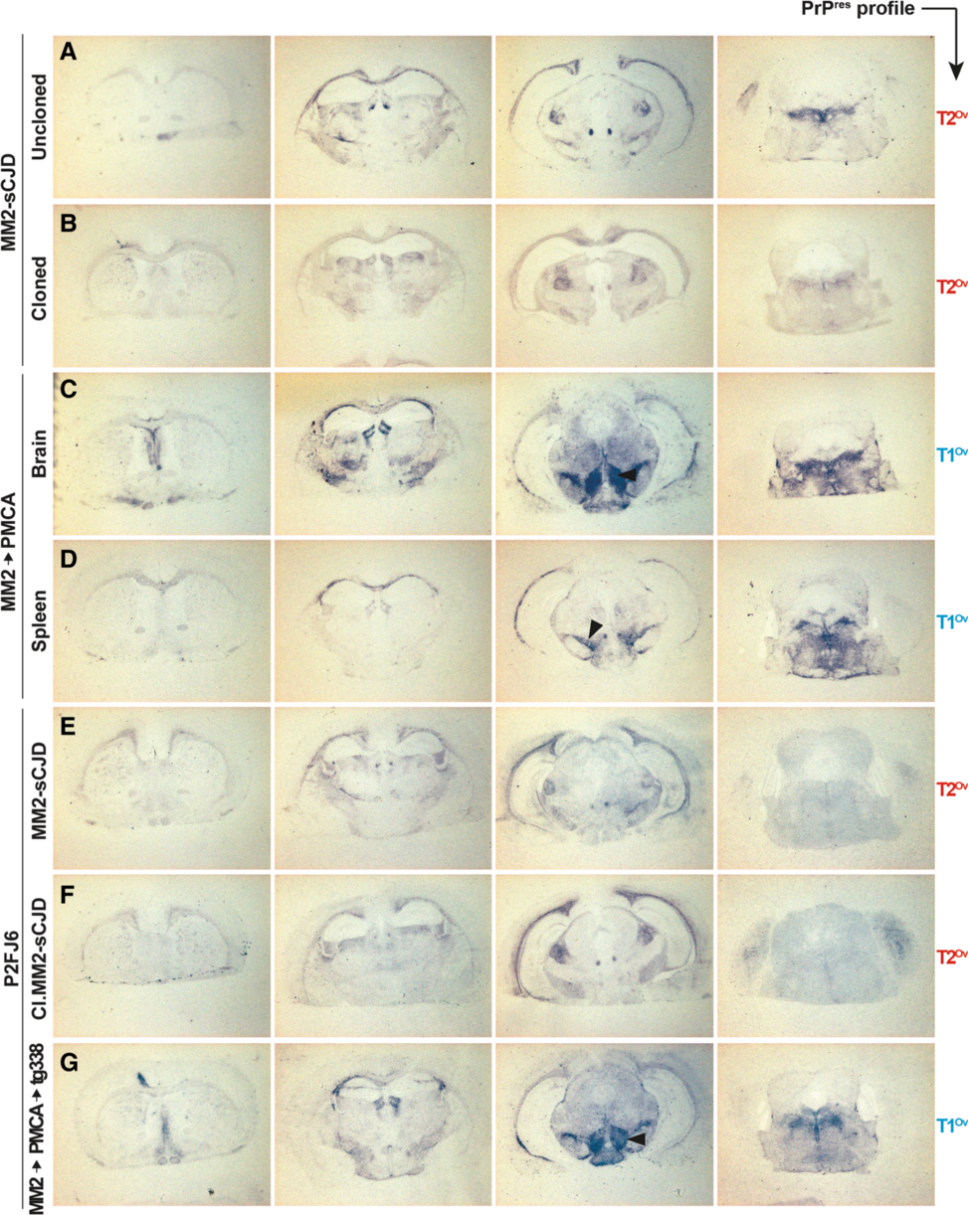
PrP^res^ deposition pattern in the brain of ovine PrP mice challenged with MM2-sCJD prions. Representative histoblots in 4 different antero-posterior sections of tg338 mouse brain after inoculation with tg338-passaged MM2-sCJD prions (**a**, 4^th^ passage), tg338-cloned MM2-sCJD prions (**b**), brain (**c**) and spleen (**d**) of tg338 mice inoculated with PMCA-amplified MM2-sCJD prions, P2FJ6 cells challenged with uncloned (**e**), cloned (**f**) and PMCA-derived (**g**) MM2-sCJD prions (MM2 → PMCA → tg338). Note the marked deposition of PrP^res^ in the lateral hypothalamic areas of mice inoculated with T1^Ov^ prions (black arrowhead). Blots were probed with 12F10

Histoblot analyses revealed preferential distribution of PrP^res^ in the dorsal and habenular, medial geniculate thalamic nuclei, in the cingulum, in the fasciculus retroflexus, in the external capsule and in the raphe nuclei regions of terminally sick tg338 brains (Fig. 3a).

Spleens from diseased mice gradually accumulate detectable levels of PrP^res^ over subpassaging (0/5 at 1^st^ passage; 1/2 on 2^nd^ passage, 5/5 on 3^rd^ and subsequent passages). The spleen-banding pattern was homogeneous and distinct from that observed in the brain, the unglysosylated PrP^res^ core migrating at ~21 kDa (Fig. 2a-b). This pattern was denominated T1^Ov^.

MM2-sCJD prions passed twice in tg338 mouse brains (T2^Ov^) were biologically cloned by end-point dilution in tg338 mice. Based on clinical signs and presence of PrP^res^ in the brain, a 100% attack rate was observed until the 10^−5^ dilution. The limiting dilution established at 10^−6^ (Table 2). Applying the Spearman-Kärber method to the number of animals positive at each dilution provided an infectious titer of 10^8.8^ ID_50_ IC in tg338/g. Subpassaging with brain material of a terminally sick mouse inoculated at the limiting dilution induced disease in 79 ± 2 days (6/6 mice). While the T2^Ov^ PrP^res^ pattern was conserved in the brain of these animals, the T1^Ov^ signature in the spleen was lost; two-third of the spleens analyzed accumulated low levels of T2^Ov^ PrP^res^ and one-third were negative (Fig. 2b). On a further subpassage with brain material, the T2^Ov^ PrP^res^ signature was observed in the 6 spleens and brains analyzed (Fig. 2b, Table 3). This material was designated tg338-cloned MM2-sCJD prions. There was limited variation in the distribution of PrP^res^ deposits in the brains of tg338 mice infected with cloned and uncloned MM2-sCJD material. PrP^res^ deposition was more and less pronounced in the dorsal thalamic nuclei and in the external capsule/cingulum, respectively (Fig. 3b).

**Table 3:** Transmission of MM2-sCJD prions to ovine PrP mice after PMCA amplification or passage through P2FJ6 cells

Inoculum	Passage	Mean incubation time^a^ (n/n_0_)	PrP^res^ pattern^b^	
			Brain	Spleen
MM2-sCJD	5	80 ± 1 (6/6)	T2^Ov^	T1^Ov^
Cloned MM2-sCJD^c^	2	79 ± 1 (6/6)	T2^Ov^	T2^Ov^
MM2-sCJD → PMCA	1	105 ± 1 (5/5)	T1^Ov^	T1^Ov^
MM2-sCJD → PMCA → tg338 brain	2	84 ± 2 (6/6)	T1^Ov^	T1^Ov^
MM2-sCJD → PMCA → tg338 spleen	2	93 ± 2 (6/6)	T1^Ov^	T1^Ov^
MM2-sCJD → P2FJ6 cells	1	80 ± 1 (6/6)	T2^Ov^	T1^Ov^
Cloned MM2-sCJD → P2FJ6 cells	1	75 ± 2 (6/6)	T2^Ov^	neg
MM2-sCJD → PMCA → P2FJ6 cells	1	118 ± 1 (6/6)	T1^Ov^	T1^Ov^
MM2-sCJD → RK13 cells → tg338	1	> 400 d (0/6)	neg	neg

n/n_0_: number of mice with neurological disease and positive for PrP^res^ in the brain by immunoblotting/number of inoculated mice

^a^Days ± SE of the mean.

^b^ T1^Ov^ and T2^Ov^ refers to the migration pattern of unglycosylated PrP^res^ at ≈ 21 kDa and ≈ 19 kDa (in the brain), respectively.

^c^tg338-MM2-sCJD prions were cloned by end-point titration in reporter tg338 mice and sub-passed.

Brain was used for inoculation unless mentioned

Neg: negative

Collectively, these results demonstrate that the serial transmission of MM2-sCJD prions to tg338 mice led, after an abrupt shortening of the incubation time, to the isolation of two strain components, a dominant T2^Ov^ component in the brain and a T1^Ov^ subcomponent that preferentially populates the spleen. Biological cloning of brain material allowed elimination of the T1^Ov^ component, at least to levels below detectable replication in tg338 mice.

### Preferential selection of the T1^Ov^ substrain component by protein misfolding cyclic amplification

**Fig. 4:**
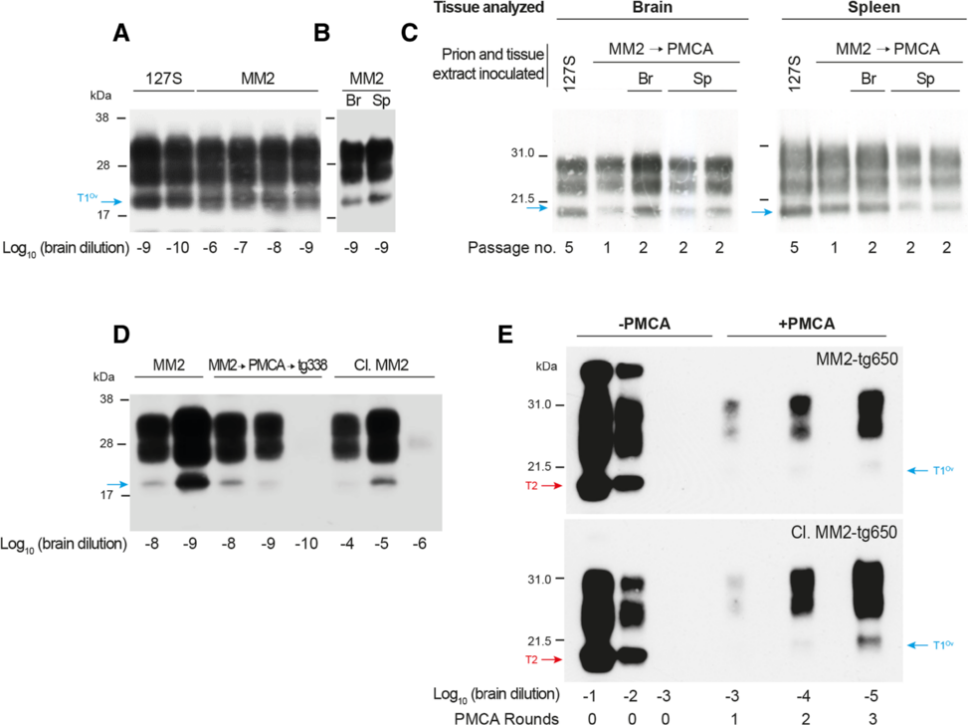
Amplification of MM2-sCJD prions by PMCA, using ovine PrP^C^ as substrate. (**a, b**) Endpoint titration of tg338-passaged MM2-sCJD prions by a single round of PMCA. Brain (**a**) or spleen (**b**) homogenates from tg338 mice infected with MM2-sCJD prions were serially diluted in tg338 healthy brain lysate, as indicated, and submitted to one round of PMCA. Each amplified dilution was analyzed by immunoblotting (Sha31 antibody) for the presence of PrP^res^ and characterization of electrophoretic pattern. Amplification of 127S prion seeds (21 kDa-PrP^res^) has been done in parallel for comparison [41]. (**c**) PrP^res^ banding pattern in the brain and spleen tissue of tg338 mice inoculated with PMCA amplified products from tg338-passaged MM2-sCJD prions (10^−8^ dilution seed) and on subpassage (MM2 → PMCA). On 2^nd^ passage, mice were challenged with either brain (Br) or spleen (Sp) extracts. The equivalent of 1 and 2 mg of brain and spleen tissue was loaded on the SDS-PAGE gels (Sha31 antibody). 127S PrP^res^ is shown as control. (**d**) Endpoint titration by PMCA of uncloned (MM2) and cloned (Cl.MM2) tg338-passaged MM2-sCJD prions. The limiting dilution achieved was compared to that observed after PMCA reactions seeded with brain homogenates from tg338 mice inoculated with PMCA-amplified MM2-sCJD prions (2^nd^ passage; MM2 → PMCA → tg338). (**e**) Amplification of human MM2-sCJD prions by PMCA, using tg338 healthy brain lysate as substrate. PMCA reactions were seeded with 10^3^-diluted brain material from human PrP tg650 mice infected with uncloned (3^rd^ passage; MM2-tg650) or cloned (4^th^ passage; Cl.MM2-tg650) Three amplification rounds were performed. After each round, the amplified products were analyzed for PrP^res^ content and electrophoretic pattern by immunoblot (Sha31 antibody) or diluted 1:10 in fresh substrate to seed the following round

Having identified that tg338-adapted, MM2-sCJD prions were a mixture of two components, we next examined whether one would outcompete the other in cell free conversion assays such as PMCA [52]. PMCA allows PrP^Sc^ templating the conversion of PrP^C^ by repetitive cycles of incubation and sonication, leading to amplification of subinfectious levels of PrP^Sc^. Brain homogenates from tg338-passaged MM2-sCJD prions (2^nd^ or 3^rd^ passage; T2^Ov^ signature) were serially diluted up to the 10^−12^ dilution and mixed with healthy brain homogenate from tg338 mice. The resulting mixture was submitted to one round of PMCA reaction, using a highly efficient method previously described [41]. The amplified products were then PK digested and analyzed by Western blot. Detection of PrP^res^ was achieved in reaction mixtures seeded with 10^−9^-diluted brain material (n = 6 independent experiments), suggesting efficient amplification, as previously observed with 127S scrapie prions (Fig. 4a, d, [41]). Remarkably, the banding pattern of all amplified products (even after seeding with lowly-diluted brain material) was distinct from that of the inoculum, the unglysosylated PrP^res^ core migrating at ~21 kDa (Fig. 4a), thus suggesting that the T1^Ov^ subcomponent was preferentially amplified. PMCA performed with T1^Ov^-enriched tg338 spleen material (at 3^rd^ passage) further confirmed the efficient amplification of this prion component. The limiting dilution established at 10^−9^, as with brain material (n = 3 independent experiments) and the PrP^res^ profile of the amplicons appeared as T1^Ov^ (Fig. 4b).

To ascertain whether the PMCA process results in the selective amplification of T1^Ov^ prions, tg338 mice were intracerebrally challenged with products generated from the 10^−8^ brain seed, that is 100-fold below the lowest dose resulting in positive transmission by bioassay (Table 2). Tg338 mice succumbed with disease in 105 ± 1 days, a survival time that established to approximately 90 days on further subpassaging (Table 3). A unique T1^Ov^ type electrophoretic signature was observed in both brains and spleens over these two passages, whether subpassage was performed with brain or spleen material (Fig. 4c, Table 3). After inoculation of brain or spleen material, PrP^res^ deposition in the brain also differed from that observed with non amplified MM2-sCJD prions. PrP^res^ deposited more ventrally in the lateral hypothalamic areas and granular-like deposits were observed in the corpus callosum (Fig. 3 c-d). This material was designated PMCA MM2-sCJD prions. Brains infected with PMCA MM2-sCJD prions were also submitted to PMCA reaction. The seeding activity detection limit established at the 10^−9^ dilution, as observed with uncloned MM2-sCJD prions passaged in tg338 mice (Fig. 4d).

The use of tg338-cloned MM2-sCJD brain material further demonstrated that T2^Ov^ seeds were poor convertors in PMCA reaction. The limiting dilution achieved was dramatically decreased by 10^4^-fold as compared to uncloned MM2-sCJD prions (Fig. 4d). PrP^res^ banding pattern observed was still T1^Ov^, suggesting that one biological cloning was not sufficient to eliminate totally this subcomponent for PMCA reaction, owing to the improved sensitivity of this technique [41].

Collectively, these data indicated that the T1^Ov^ subtype was an isolable substrain component of MM2-sCJD in tg338 mice, which carried most if not all the PMCA activity. Separately, the T1^Ov^ subcomponent was pathogenic in tg338 mice, as T2^Ov^.

### PMCA-generation of the T1^Ov^ substrain component by seeding ovine PrP^C^ with human MM2-sCJD prions

PMCA has been shown as a convenient model to investigate experimentally prion species barrier [3, 18, 25]. We thus examined the outcome of confronting directly human MM2-sCJD prions to ovine PrP^C^. Brain homogenates from uninfected tg338 mice were seeded with a 10^−3^ dilution of brain homogenate from tg650 mice infected with uncloned or cloned MM2-sCJD prions. To augment the chance of crossing the species barrier, up to three rounds of PMCA were performed. At each round, the amplified products were analyzed for PrP^res^ content by Western blot. As shown in Fig. 4e (representative of n = 4 independent experiments), PrP^res^ was detected at each round. The electrophoretic pattern observed resembled T1^Ov^, the unglysosylated PrP^res^ core migrating at ~21 kDa. A T2^Ov^ PrP^res^ signature was occasionally observed in the PMCA products (1 out of 16 positive reactions, data not shown), suggesting possible stochastic generation of these prions on the ovine PrP sequence. Remarkably, the same efficacy of amplification relative to the number of rounds was found with either cloned or uncloned tg650 material, allowing detection of T1^Ov^ PrP^res^ from 10^5^-fold diluted seeds in three rounds (Fig. 4e). It can be extrapolated from these data that under the constraint of having to be amplified by PMCA with an ovine PrP substrate, T1^Ov^ prions can directly emerge from a human MM2-sCJD prions population, cloned or not.

### The two MM2-sCJD ovine subcomponents can be stably passaged in a Rov cell subclone

RK13 cells expressing the VRQ allele of ovine PrP are permissive to ‘fast’ scrapie prions, serially passaged or not in tg338 mice [32, 56, 58]. Motivated by the short incubation times of MM2-sCJD prions in tg338 mice, we examined whether they could propagate in these cells and whether one subcomponent would compete out the other. The Rov9 cell clone [58] and congeners were poorly permissive to tg338-passaged MM2-sCJD prions. Cells mostly failed to accumulate detectable levels of PrP^res^, despite exposure to lowly diluted brain homogenate, and prolonged incubation of the cells with the inoculum (data not shown and Munoz-Montesino et al., submitted for publication). Another Rov construct constitutively expressing ovine PrP (P2FJ6 cells) and used routinely in our scrapie cell assays because of its marked susceptibility to scrapie 127S prions ([32, 56] and to be published elsewhere) was permissive to MM2-sCJD prions. To sustain replication, it was necessary to adapt the infection protocol, so as to allow longer time of exposure between the cells and the inoculum (see methods).

**Fig. 5:**
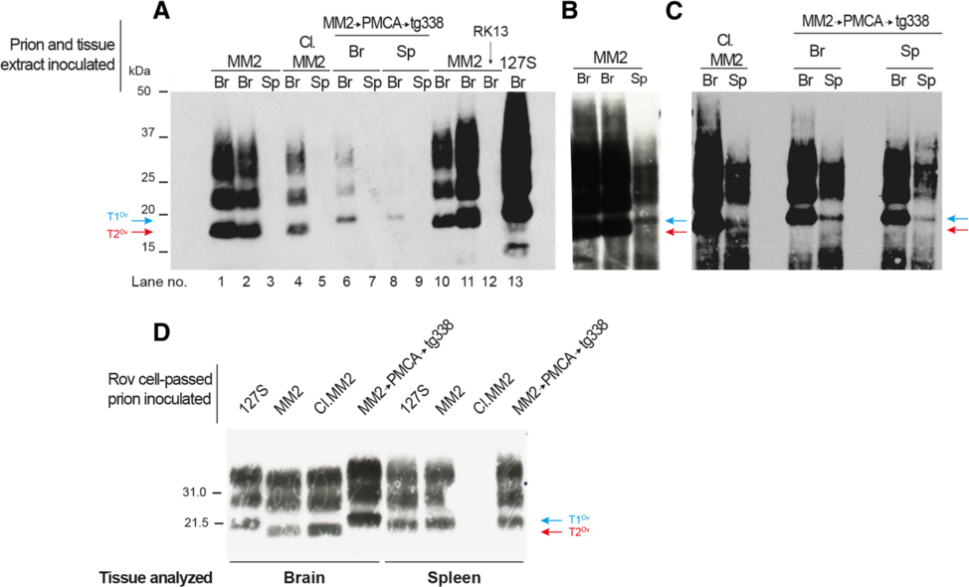
Infection of P2FJ6 cells with ovine MM2-SCJD prions, and PrP^res^ glycopattern of the cell-passaged prions in tg338 mice. (**a-c**) Western blot analysis of PrP^res^ in P2FJ6 cell lysates infected with brain (Br) and spleen (Sp) extracts of tg338 mice infected with MM2-sCJD prions, either uncloned (MM2) or cloned (Cl.MM2), and mice challenged with either brain or spleen extracts of PMCA MM2-sCJD prions (MM2 → PMCA → tg338). The results are shown over 3 (**a**) or 4 passages (**b**, **c**) post-cell exposure, except in lanes 11-12 in (**A**), where the analysis was made at 14 passages. The gels in **b** and **c** are overexposed purposely to reveal accumulation of T1^Ov^ PrP^res^ after infection with spleen material. Untransfected RK13 cells (RK13, arrow in **a**) did not accumulate detectable levels of PrP^res^. Infection with 127S prions was performed in parallel to estimate the efficacy of infection. The gels were loaded with 250 μg of PK-digested protein or 80 μg for 127S infection. The blots were probed with Sha31 antibody. (**d**) Western blot analysis of PrP^res^ in the brain and spleen tissue of tg338 mice challenged with P2FJ6 cell lysates infected with uncloned (MM2), cloned (Cl.MM2) MM2-sCJD prions, PMCA MM2-sCJD prions (MM2 → PMCA → tg338), and 127S as control. The equivalent of 1 and 2 mg of brain and spleen tissue was loaded on the SDS-PAGE gels. The blots were probed with Sha31 antibody

Infection of P2FJ6 cells with brain extracts from tg338-adapted MM2-sCJD prions led to detection of PrP^res^ from 2 cell passages onwards, as shown by Western blot analyses. The signal increased with passaging and was detected up to 14 passages post-infection, i.e. 14 weeks pi (Fig. 5a). The banding pattern observed was a T2^Ov^ pattern with regard to the migration size of unglycosylated PrP^res^. The number of passages of MM2-sCJD prions onto tg338 mice before infection of the P2FJ6 cells was without influence on the efficacy of the infection, and on the PrP^res^ electrophoretic pattern (data not shown). PrP^res^ could not be detected in the parental RK13 cell line exposed to tg338-MM2-sCJD prions (Fig. 5a), indicating that the signal observed was not remnant inoculum. Of note, the overall levels of PrP^res^ in the P2FJ6 culture were below those observed after infection (at the same dose) with 127S scrapie prions (Fig. 5a). Exposing P2FJ6 cells to T1^Ov^-enriched spleens of tg338 mice infected with MM2-sCJD prions led to accumulation of low levels of PrP^res^, with a T1^Ov^ banding pattern (Fig. 5b), suggesting that the cells were also permissive to T1^Ov^ prions.

All tg338 mice inoculated with P2FJ6 cell lysates exposed to tg338 brain-passaged MM2-sCJD prions (at passage 7, to exclude remnant inoculum, as confirmed with RK13 cells) developed disease with a strain phenotype similar to MM2-sCJD prions in tg338 mice: the mean incubation time established at 80 dpi (Table 3); brain PrP^res^ exhibited a T2^Ov^ banding pattern (Fig. 5d), and neuroanatomical distribution of PrP^res^ closely resembled that observed upon direct MM2-sCJD subpassaging (Fig. 3e). Immunoblot examination of the spleens revealed the presence of the T1^Ov^ component (Fig. 5d), suggesting ‘silent’ propagation in the P2FJ6 cells.

To further ascertain that P2FJ6 cells could propagate T1^Ov^ as T2^Ov^ prions and exclude any in-cell reversal phenomenon between the two components, P2FJ6 cells were exposed to the two T2^Ov^ and T1^Ov^ subcomponents separately, by using brain material derived from tg338-cloned MM2-sCJD prions (i.e. enriched in T2^Ov^) or from PMCA MM2-sCJD prions (i.e. enriched in T1^Ov^), respectively. In that case, cells faithfully accumulated prions with T2^Ov^ and T1^Ov^ PrP^res^ signatures, respectively (Fig. 5a). When cells were challenged with spleen material from the same mice, infection proceeded at slower rate, as shown by the lower levels of PrP^res^ accumulation, which necessitated highly exposed gels to be visible (Fig. 5c). The PrP^res^ signature observed were T2^Ov^ from cloned material and T1^Ov^ from PMCA passaged material (Fig. 5c), as in the spleen. Whatever the tissue used to infect the cells, the T1^Ov^ and T2^Ov^ signatures were kept up to 12 cell passages, suggesting stable cell replication of the two subcomponents independently (data not shown). Reinoculation of the so-called T2^Ov^ and T1^Ov^ cell lysates to tg338 mice confirmed the faithful and separate propagation of the two agents in the cells. Indeed their strain specific signature was preserved after intermediate passage in cells, with regard to incubation time (Table 3), PrP^res^ banding pattern in brain and spleen tissues (Fig. 5d) and histoblot analyses (Fig. 3f-g). Altogether, these data indicate that Rov P2FJ6 cells were fully competent to replicate the two tg338 passaged MM2-sCJD infectious components.

## Discussion

We report here on the capacity of a rare subtype of human sCJD prions (MM2, cortical variant) to adapt onto the ovine PrP sequence, thereby modifying its apparent substrain composition and tissue tropism. We further show that the isolated variants can exhibit distinct replicative or biological behavior depending on the matrix or environment surrounding the prion conversion process.

Human PrP tg650 mice challenged with the MM2-cortical CJD subtype developed a clinical disease at full attack rate in less than 300 days. A T2 PrP^res^ profile was invariably observed in the inoculated mice. These characteristics were maintained over passaging, suggesting faithful propagation of this agent in this mouse line. Together, the incubation time in tg650 mice and the brain PrP^res^ profiles were unique among the panel of CJD cases transmitted so far to the tg650 line (Fig. 1 and unpublished data), thus fully supporting the contention that MM2-cortical CJD is a specific CJD subtype, as previously concluded from the absence of transmission to transgenic lines expressing physiological level of human PrP [10, 30, 40]. Human PrP overexpression in tg650 mice may have been key in the success of transmission. Additionally, we used a cerebellum extract as inoculum, whereas previously, a cerebral cortex extract was used. Different brains and different brain regions harbor prion infectivity titers that can vary by 100-fold with regard to the number of infectious units per milligram of tissue [1, 11]. While such a difference would barely affect the transmission rate of MM2-sCJD prions to tg650 mice (Table 2), this might be an issue in knock-in human PrP transgenic mice in which disease develops at slower pace [10].

While MM2-sCJD prions propagated faithfully in human PrP mice, the outcome was different upon transmission to ovine PrP tg338 mice. Efficient transmission occurred on primary passage, at near full attack rate, albeit with a prolonged incubation period. A drastic reduction in the incubation time from 550 to 80 days occurred on secondary passage, consistent with the isolation of a variant, or a ‘mutant’ by analogy to mutational events observable with conventional microorganisms [2, 4, 5, 8, 9, 13, 29, 46, 47, 54]. While a 19K-like PrP^res^ signature predominated in tg338 mouse brains as in human or human PrP mouse brain, a second signature, designated T1^Ov^ gradually emerged with subpassaging in tg338 mouse spleens. This distinct tissue tropism and the possibility to isolate, by conventional biological cloning or cell-free amplification, and further propagate separately and with fidelity the T2^Ov^ and T1^Ov^ types in tg338 mice or P2FJ6 cells indicate that a pair of *bona fide* prion strains has been isolated on the ovine PrP sequence.

Once propagated alone, we failed to evidence any gradual shift of T2^OV^ towards T1^Ov^ prions in tg338 mice or in P2FJ6 cells, suggesting that the interspecies prion conversion events have been instrumental in the emergence of the T1^Ov^ substrain component. A recurrent and often perplexing question [8, 21, 60, 61] is whether these agents have been generated *de novo* on confrontation to the new PrP sequence or have been preferentially selected from pre-existing PrP^Sc^ conformations. Our data support the contention that T1^Ov^ prions have been generated *de novo*: First, if the T1^Ov^ agent preexisted in the human MM2-sCJD source, it was not able to replicate at detectable levels in the spleens of tg650 mice, at variance with other human prion strain sources [5, 6]. Second, we found no evidence of a T1 signature in the brain of tg650 mice inoculated with MM2-sCJD prions that may putatively be at the origin of T1^Ov^ prions onto the ovine PrP sequence (Fig. 1). Third, T1^Ov^ prions could be directly generated by PMCA from the human MM2-SCJD prions population passaged in tg650 mice. The lack of obvious difference in the PMCA efficacy between uncloned and cloned material (Fig. 4e), the latter being theoretically less populated in a potential T1^Ov^ subcomponent (to be compared with the dramatic reduction in the PMCA efficacy when tg338-passaged uncloned and cloned MM2-sCJD prions are amplified, Fig. 4d), suggests *de novo* generation of T1^Ov^ on confrontation to the ovine PrP sequence. We acknowledge that definitive proof is lacking and it is possible that substrain heterogeneity may constantly arise even in cloned populations during propagation [39].

Our observations are adding to the view that 2 distinct prion strains can propagate in neural and extraneural tissues of the same host individual [5, 7]. We are currently comparing the retro-transmission properties of the T1^Ov^ and T2^Ov^ types to human PrP mice to examine whether the selection pressure imposed by the heterologous transmission was lower in spleen than brain tissues from tg338 mice, as previously observed [5]. The situation may however be drastically different here, as the T1^Ov^ component emerged gradually in the spleen with subpassaging and adaptation, whereas previously, the spleen component emerged first, and at higher rate, than the brain component [5].

The replicative properties of the two MM2-sCJD ovine prions markedly differed depending on the environment/matrix where ovine PrP conversion occurred: the two components fairly propagated in tg338 mice (yet at variable, tissue-dependent levels) and in P2FJ6 cells, whereas the T1^Ov^ component was selectively amplified by PMCA, despite the use of tg338 mouse brain as PrP^C^ substrate in the PMCA reaction. Comparative (ongoing) titration of isolated T1^Ov^ and T2^Ov^ prions suggest that both agents exhibit similar infectious titers (Table 4). Serial passaging in P2FJ6 cells also indicates that none of the component was outgrowing the other over passaging, suggesting roughly similar half-life and doubling time. Despite the point that our PMCA protocol is relatively promiscuous, allowing efficient amplification of several strains from different species [41], it remains possible that T2^Ov^ amplification necessitates very specific PMCA conditions. Incidentally, PMCA appears as a convenient, alternative method to biological cloning, to isolate one prion substrain component from a mixture.

**Table 4:** Endpoint titration of isolated T1^Ov^ and T2^Ov^ MM2-sCJD prions in ovine PrP mice

Inoculum	Dilution	Mean incubation time^a^ (n/n_0_)	
		T2^Ov^ ^b^	T1^Ov^ ^b^
MM2-sCJD	10^−4^	142 ± 8 (5/5)	nd
	10^−5^	146 (1/6)	148 ± 4 (6/6)
	10^−6^	174; 192 (2/6)^c^	212 ± 6 (3/6)^c^
	10^−7^	> 450 (0/6)^c^	> 450 (0/6)^c^
	10^−8^	>450 (0/6)^c^	> 450 (0/6)^c^

n/n_0_: number of mice with neurological disease and positive for PrP^res^ in the brain by immunoblotting/number of inoculated mice

^a^Days ± SE of the mean.

^b^The T2^Ov^ and T1^Ov^ brain material were from tg338-cloned MM2-sCJD prions and from PMCA MM2-sCJD prions, respectively.

^c^ongoing experiment

nd: not done

Within the quasi-species concept applied to prions, -which proposes that prions are not constituting a single clone but are embedded with all PrP^Sc^ conformational variants [21, 61]-, our observations would suggest that there has been a bottleneck event in tg338 mice (due to the heterologous PrP transmission) which has affected MM2-sCJD prions fitness, at least on primary passage, and has led to the emergence of two strain components. Key to the quasi-species definition is the existence of intra-population interactions, either complementation or interference [44]. Complementation is difficult to accommodate with the observation that there is so far no apparent regeneration of the two T1^Ov^ and T2^Ov^ subcomponents after intermediate separation of one of the component, either by PMCA or biological cloning. As individuals, both components do not harbor different fitness with regard to incubation time, cell replicability and infectious titer; however, jointly, T2^Ov^ markedly outcompetes T1^Ov^ in either P2FJ6 cells or in tg338 brain. Neuroanatomically, the absence of replication of T1^Ov^ prions in certain brain target areas, that are free of T2^Ov^ prions (such as the lateral hypothalamic area (Fig. 3)) suggest local interfering mechanisms, independent of competition for the same PrP substrate [55]. We thus propose that the MM2-sCJD ‘mutant’ isolated in tg338 mice is not a mere agglomeration of independently acting T1^Ov^ and T2^Ov^ conformations.

Since the discovery that RK13 cells expressing the ovine PrP VRQ allele were permissive to certain scrapie prions [23, 58], we and others have made numerous attempts to infect these cells or more permissive clones such as the P2FJ6 one with ovine prions sources, passaged or not onto tg338 mice. Most attempts have failed, except for prions classified as ‘fast’, as based on their short incubation time in reporter tg338 mice (e.g. 127S and LA21K *fast*, [42, 56]). Here we show that two other relatively ‘fast’ agents originating from MM2-sCJD can fairly propagate in Rov cells at their maximum infectivity levels, as assessed by the tg338 bioassay. Many factors can account for the difficulties to replicate prions in immortalized cell lines. Obviously, prion-doubling time must surpass the cell division rate. For ‘fast’ strains such as 127S and LA21K *fast*, it appears that a subset of small oligomers are by far the most active with respect to prion replication in vivo and to seeding activity by PMCA [32]. This might be key to their sustained ability to replicate in cells. We are currently investigating the oligomeric state of the most active sCJD aggregates. In any case, this extended panel of strains replicating in cell culture opens the possibility to compare their biology in cells expressing wild type or mutated forms of PrP (Munoz-Montesino et al., submitted for publication).

## Conclusions

We report here that prions associated with a cortical subtype of human MM2-sCJD prions converts the ovine VRQ PrP in its disease-associated form. The same source propagates efficiently in mice expressing the ARQ allele of ovine PrP ([43]). Our ongoing studies indicate that human MM1 and MV2 CJD prions can also adapt on the VRQ allele of ovine PrP. This indicates that CJD prions exhibit a retro-zoonotic potential. Reversely, certain scrapie prions could propagate on human PrP mice [17] and in non-human primates which exhibit similar amino acid sequence as humans [22]. Thus the human to ovine prion transmission barrier may not be absolute. From an evolutionary point of view, these data interrogate on the potential interplay between animal and human prions and their true origin, either spontaneous/sporadic versus infectious. Their fate may not be not as compartmentalized as commonly believed.

## Additional file

Additional file 1: **Supplementary Material.** (PDF 2698 kb)
