# Supplementary material for: Emergence of two prion subtypes in ovine PrP transgenic mice infected with human MM2-cortical Creutzfeldt-Jakob disease prions
Source: Acta Neuropathol Commun. 2016 Feb 5;4:10. doi: 10.1186/s40478-016-0284-9 (PMC4743415; doi:10.1186/s40478-016-0284-9)
Supplement: Supplementary file 1 — Supplementary Material. (PDF 2698 kb) [file 40478_2016_284_MOESM1_ESM.pdf]

Supplementary Material

Emergence of two prion subtypes in ovine PrP transgenic mice infected with human MM2-cortical Creutzfeldt-Jakob disease prions

Jérôme Chapuis, Mohammed Moudjou, Fabienne Reine, Laetitia Herzog, Emilie Jaumain,

Céline Chapuis, Isabelle Quadrio, Jacques Boulliat, Armand Perret-Liaudet, Michel Dron,

Hubert Laude, Human Rezaei, and Vincent Béringue

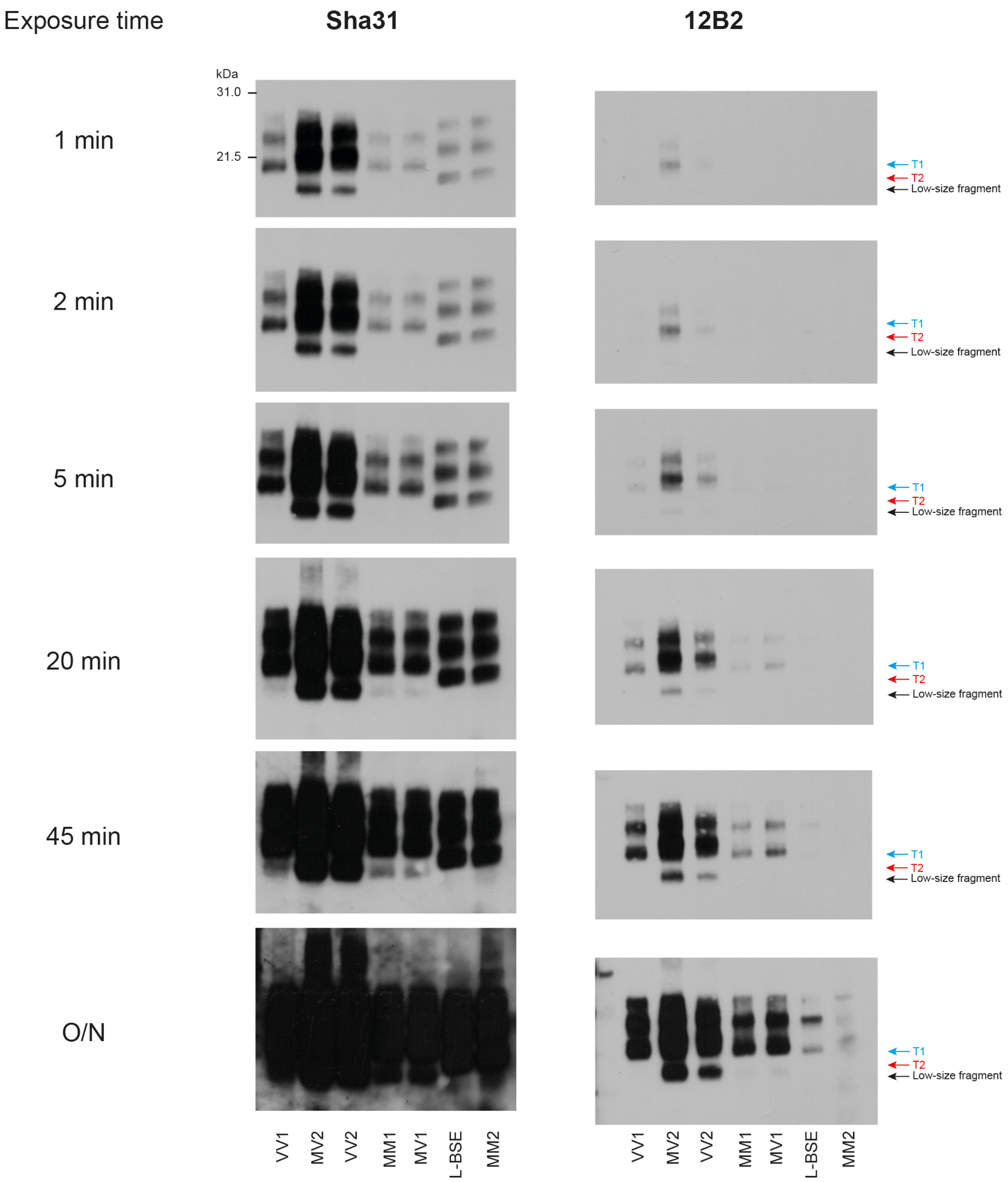

Figure S1. Electrophoretic pattern of PrP^{res} in the brain of human PrP transgenic mice inoculated with MM2-sCJD prions

Western blot analysis of PrP^{res} in the brain of tg650 mice infected with MM2-sCJD prions, after blotting with Sha31 antibody (left) or 12B2 antibody specific for Type 1 PrP^{res} (right), at different times of exposure. The banding patterns observed on transmission of other CJD

subtypes and atypical L-BSE (which exhibits a T2 signature) are shown for comparison. The equivalent of 1 mg (Sha31) and 7 mg (12B12) tissue were run on the SDS-PAGE gels for MM2-sCJD and L-BSE infected brains. The equivalent of 1 mg (Sha31, VV1, MM1, MV1; 12B2, MV2, VV2), 0.5 mg (Sha31 MV2, VV2), 2 mg (12B2, VV1, MM1, MV1) were loaded for the other samples.

Red and blue arrows denote the unglysosylated bands of PrP^{res} migrating at 21 kDa (T1) and 19 kDa (T2), respectively. A black arrow shows the presence of low-size PrP^{res} fragments in the brain of diseased tg650 mice. Molecular masses (MM) of protein standards are indicated in kilodaltons.
